# Monomeric C-Reactive Protein: Current Perspectives for Utilization and Inclusion as a Prognostic Indicator and Therapeutic Target

**DOI:** 10.3389/fimmu.2022.866379

**Published:** 2022-03-04

**Authors:** Mark Slevin, Nima Heidari, Leonard Azamfirei

**Affiliations:** ^1^ Department of Life Sciences, George Emil Palade University of Medicine, Pharmacy, Science and Technology, Targu Mures, Romania; ^2^ The Regenerative Clinic, London, United Kingdom; ^3^ The School of Life Sciences, Manchester Metropolitan University, Manchester, United Kingdom

**Keywords:** monomeric C-reactive protein, diagnostic, therapeutic, cardiovascular disease, neuropathology

## Abstract

Monomeric C-reactive protein (mCRP), once thought to be a figment of the imagination and whose biological activity was ascribed to its sodium azide preservative, has now pronounced itself as a critical molecule playing a direct role in mediating many of the acute and chronic aberrant pathological responses to inflammation. In this focused mini review, we describe the currently attributed pathobiological interactions of mCRP in disease, where its tissue and cellular distribution and deposition have recently been clearly characterized and linked to inflammation and other pathway-associated progression of neurological and cardiovascular complications and deleterious outcomes. and focus upon current opinions as to the diagnostic and prognostic potential of mCRP-plasma circulating protein and define the possible future therapeutics including ongoing research attempting to block CRP dissociation with small molecule inhibitors or prevention of cell surface binding directly using antibodies or modified orphan drug targeting directed towards CRP, inhibiting its cellular interactions and signaling activation. There is no doubt that understanding the full influence of the biological power of mCRP in disease development and outcome will be considered a critical parameter in future stratified treatment.

## Introduction

The physiological functions of C-reactive protein (CRP) and the precise mechanisms through which it activates complement, modulates the innate immune system, and opsonizes invading pathogens are fully understood to date (1) and therefore, this mini-review will focus on summarizing the emerging pathological indications of its precursor sub-unit-monomeric CRP (mCRP) and discussing the relevant prognostic, diagnostic and therapeutic possibilities of its utilization. Substantial increases in soluble circulating native CRP occur, following immune cell-mediated liver stimulation by interleukin-6 (IL-6), in individuals with infection and tissue damage, and may result from an acute insult such as sepsis, myocardial infarction or stroke or from chronic conditions (with prolonged elevation) including a variety of autoimmune diseases (2).

The blood insoluble mCRP, (its dissociated and highly biologically active form), is produced in inflammatory environments following lyso-phosphatidylcholine recognition and subsequent Fc-Gamma receptor/integrin binding with compatible ‘activated’ blood cells, platelets, circulating microvesicles and at sites of tissue damage (3). Potempa et al. eloquently summarized the process in a recent review explaining how the hepatocyte-produced pentameric blood soluble and weakly anti-inflammatory protein becomes converted to an intermediate form that is less soluble and exists as a transitional or modified pCRP bound to cell membrane exposed phosphocholine groups, prior to rapid disulfide bridge breakdown and formation of the monomeric version that is insoluble in the blood and found almost entirely partnered with particulate components, cells and tissues and having significantly higher inflammatory and other bioactive properties (4).

This mCRP dissociated form is now understood to be capable of directly and actively aggravating perpetuating inflammation at least in part through activation of M1 macrophage phenotype and subsequent expression of cytokines including tumor necrosis factor-alpha (TNF-α) and IL-6, in addition to promoting platelet and platelet-macrophage adhesion and aggregation (5). Whilst measurement of serum soluble native (high-sensitive) CRP remains valuable for monitoring general levels of inflammation and infection, it is now clear that a complete understanding of the pathobiological course of inflammatory disease requires us to assess the footprint of mCRP and consider opportunities to modify its effects and concomitantly protect patients from its deleterious activities.

## Evidence of the Disease Modifying Behavior of mCRP

Probably the most studies field is ***cardiovascular disease***. Development of atherosclerosis is a complex chronic condition, with many risk factors, occurring over several decades primarily during adult life (6). Damage to the arteries results in local inflammation which attracts immune cells to the site and over-time, creates a plaque that may be stable (fibrous) or unstable (inflammatory and vascular; and susceptible to thrombosis). Studies over the past decade or so have revealed the presence of mCRP within growing plaques (7), specifically those with an unstable phenotype, and in addition, both *in vivo* models and *in vitro* mechanistic studies have confirmed the capability of mCRP to activate signaling pathways associated with aberrant angiogenesis, promoting monocyte recruitment to the plaque, and inducing macrophage-platelet activation and aggregation that potentially triggers plaque erosion and thrombosis (8, 9).

An important point of note is that whilst more than a decade ago the existence and biological relevance of mCRP was not fully understood, a number of studies have highlighted the fact that effectively, all manufactured CRP antibodies bind to mCRP when examined/used in IHC studies. The literature therefore provides previously untold additional evidence of the protein presence within diseased tissues; for example, Sun et al. identified CRP (now recognized to be mCRP) in atherosclerotic lesions of hypercholesterolemic rabbits as well as in unstable or ruptured human coronary artery plaques (10). Recently, Siennicka (11), proposed that the extent of mCRP-binding to platelets and endothelial microvesicles might predict a weaker response to anti-platelet therapy in patients at risk of thrombosis and this could be monitored as part of a stratified precision medicine approach to treatment. This phenomenon had already been recognized and characterized by Habersberger *et al.* (12) where they discovered that microparticles obtained from the whole blood of patients with myocardial infarction contained significantly more mCRP than normal healthy control individuals; this suggested as mechanism for the transport of pro-inflammatory signaling systemically and to tissues.

Recently, Melnikov et al. (13) described significantly increased concentrations of blood circulating mCRP-bound macrophages in patients with coronary artery disease, whilst Zha et al. (14), showed increased infiltration of macrophages polarized to the inflammatory M1 phenotype (induced through the JNK signaling pathway activation) in mice following experimental coronary artery occlusion and intra-venous treatment with mCRP; and this was associated with increased myocardial infarct size, scar and fibrosis. These observations also showed the presence of mCRP in human infarcted myocardial tissue surrounding myocytes in post-mortem tissues, identified by IHC using standard manufactured CRP-binding antibodies.

Taken together all these findings can be interpreted as providing evidence of a direct role of mCRP in promoting the chronic buildup of plaque in vessel walls, potential shifting the balance to an unstable/vulnerable and inflammatory phenotype, and provision of a systemic circulating micro-environment triggering thrombotic potential.

**Figure 1:**
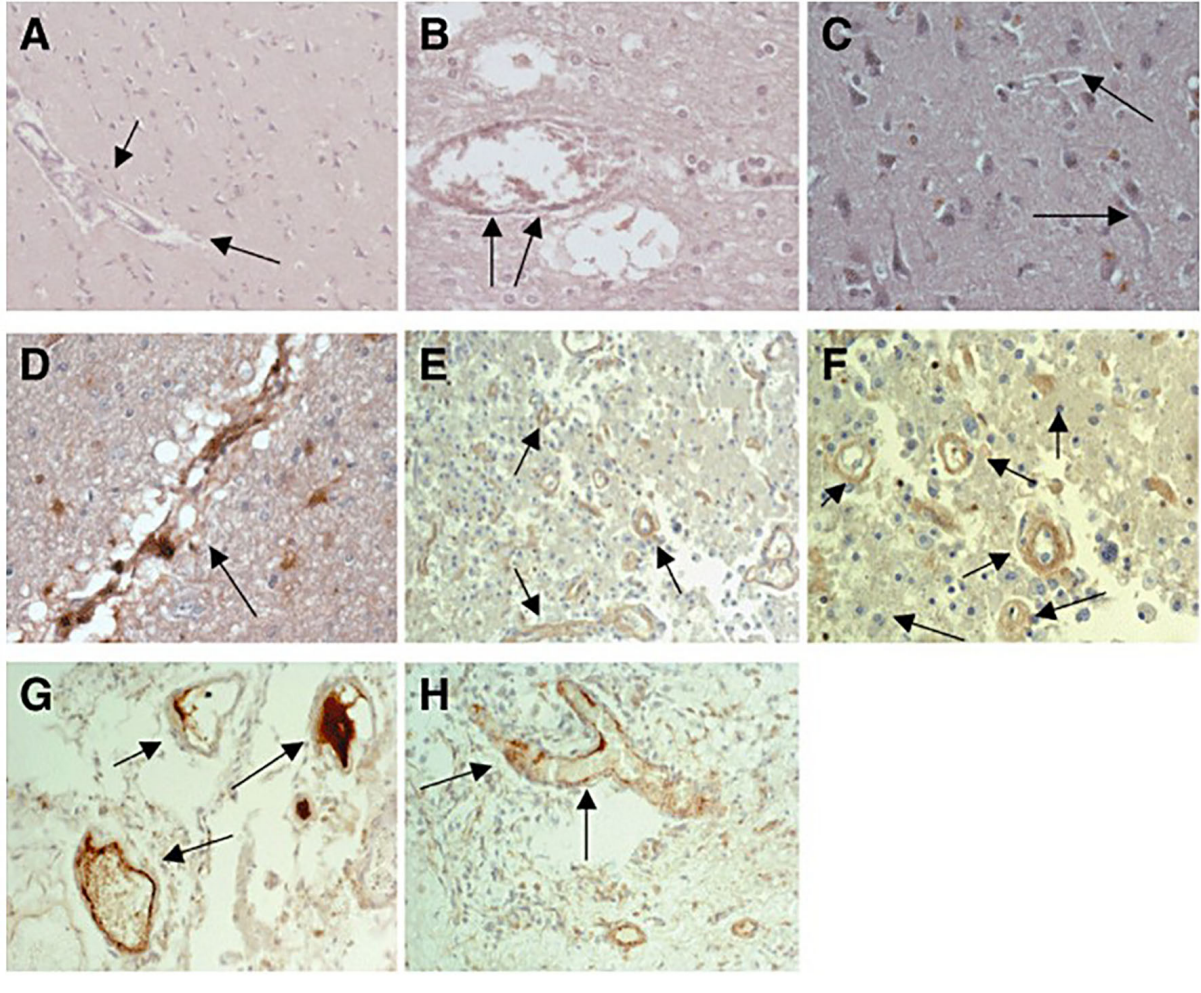
**(A)** Expression of native C‐reactive protein (nCRP) in contralateral brain tissue: almost no expression of nCRP was seen in blood vessels and neurons from normal looking brain tissue [arrows mark the blood vessels; 3,3′‐diaminobenzidine‐tetrachloride (DAB) brown development; Patient 17]. **(B)** Expression of nCRP in peri‐infarcted brain tissue again showing no notable expression, apart from occasional staining within the lumen of larger vessels (arrows; DAB brown development; Patient 17). **(C)** Expression of monomeric form of CRP (mCRP) in contralateral brain tissue: almost no expression of mCRP was seen in normal looking brain tissue (arrows mark the blood vessels; DAB brown development). mCRP was abundantly expressed in peri‐infarcted **(D–F)** and infarcted **(G, H)** brain tissue particularly in microvessels (marked by arrows; DAB brown development). Taken from Reference (16).

In ***neurological conditions*** mCRP also seems to make a significant and deleterious contribution to disease pathology. Pathological conditions involving neuroinflammation appear to encompass expression of or tissue accumulation of mCRP for example within the brain after stroke. More than a decade ago, Yu et al. (15), using antibodies recognising all forms of CRP probably identified mCRP in the infarcted regions of embolic stroke using a rabbit model of cerebral infarction. Slevin et al. (16) confirmed the presence of mCRP using specific antibodies and showed that it became particularly associated with peri-infarcted microvessels after ischaemic stroke and was also seen within the lumen of some, suggesting systemic inflammatory origin (**Figure 1**); Similarly, in hemorrhagic stroke, mCRP was observed in both microvessel and larger vessels during the chronic phase, primarily within the peri-hematoma, and associated with aquaporin-4 (associated with oedema) over-expression (17). The authors noted what appeared to be a transfer of mCRP to remote brain regions within the hypothalamus-and this was replicated in a mouse model following stereotactic hippocampal injection of mCRP. The most likely hypothesis here is that mCRP is carried within the micro-circulation perhaps piggy backed upon microvesicles or immune cells-and thus the potential impact of its primary deposition could be magnified.

Regarding longer-term effects of deposition or presence of mCRP within the brain, Strang et al. (18), in 2012 demonstrated that CRP dissociated to mCRP *in vitro*, in the presence of non-aggregated amyloid beta (42) peptide {Aβ(42)}. When they examined the frontal cortex of individuals who died with Alzheimer’s disease (AD), they discovered the presence of mCRP in cortical Aβ plaques and suggested it may have a role in amplifying local neuro-inflammation and promoting tissue degeneration. Since then, further investigations have confirmed the ability of mCRP following hippocampal injection in mice, to induce behavioral and memory/cognitive deficits (which was protected against following co-injection of a specific antibody against mCRP) whilst co-localizing with both phosphorylated Tau (p-Tau) and Aβ(42); an *in vitro* capability of mCRP to induce Tau phosphorylation and stimulate production of other AD-precursors including presenilin enhancer protein-2 and phosphorylated amyloid precursor protein (19, 20); and from an immunohistochemical study of a cohort of AD post-mortem cases, a strong co-localization of mCRP with inflammatory markers including CD68 (macrophages), nuclear factor kappa B and interleukin-1-beta (IL-1β) (21). Most recently, Zhang et al. (22), proposed a mechanism involving mCRP binding and phosphorylation of CD31 on endothelial cells that stimulated neuro-inflammation dependent on apolipoprotein E4 postulating this as a pathway leading to increased risk of AD. There is little doubt now that mCRP has an important role in stimulating brain systemic neuro-inflammation and promoting AD pathobiology, whilst a potential role in other brain or CNS/PNS disorders; including neuropsychological conditions, where mCRP has been recently highlighted as a possible indicator but this has yet to be researched in detail.

There are a number of other inflammation-driven pathological conditions that mCRP has recently been inextricably linked to. mCRP was shown to be present in drusen aggregates-the hallmark of ***age-related macular degeneration*** concomitant with an increased inflammatory activation of the retinal pigment epithelium and disruption of the outer blood-retinal barrier; mCRP was suggested to be involved in enhancing disruption and inflammation-associated progression of the disease (23). The plasma from a group of patients with ***sepsis*,** showed significantly higher levels of EVs bound to mCRP associated with enhanced IL-8 secretion compared with healthy individuals indicating a direct mechanism for perpetuation of the condition, whilst apheresis and removal of the mCRP was sufficient to reduce circulating cytokine levels (24); whilst, in autoimmune diseases such as ***lupus nephritis*,** highest levels of mCRP-antibodies in the serum was associated with worse symptomatic disease, increased expression of IL-6 and TNF-α, and clinical evidence of systemic lupus erythematosus (25). A study by Ruiz-Fernandez et al. (26), provided evidence for the first time that mCRP induced pro-inflammatory cytokine release including Il-6/8, matrix metalloproteinase-13 (MMP13) and nitric oxide synthase-2 (NOS2) in both human and murine chondrocytes suggesting a potential mechanism of enhanced cartilage catabolism in osteoarthritis.

Regarding the role of mCRP in ***cancer development and metastasis***, Potempa and colleagues have produced a recent, substantial, all-inclusive article documenting the potential involvement of CRP in these processes (4). Essentially, and perhaps somewhat surprisingly, xenograft animal models of cancer (breast adenocarcinoma, melanoma and others) have shown increased necrosis and reduced metastasis in the presence of mCRP and this tumoricidal activity is ascribed to promotion of the innate immune response and specifically macrophage activation. The promotion of ROS production and increased cytotoxic capacity could support tumor progression (27). Having previously identified that mCRP also induces aberrant angiogenesis with leaky and hemorrhagic microvessels, there is the possibility that mCRP may also contribute to a blocking of tumor vascularization and this may also account for some of its necrotic capacity/protective effects although further investigations are necessary in order to establish this hypothesis.

## Prognostic, Diagnostic, and Therapeutic Potential of Circulating mCRP

Given the almost opposing properties of nCRP versus mCRP, it seems that as biomarkers of inflammation, disease progression or recovery and prognosis stratification, a ratio of the two isoforms might glean significantly more clinical insight. Specifically, expression of the pro-inflammatory highly biologically active but blood insoluble mCRP has been quantified from the plasma as particulate or cellular-bound protein and related to disease pathophysiology. In some disease conditions, the relative circulating expression of nCRP could reflect status change or severity matching the tissue deposition of mCRP-for example, Kim *et al.* (28) found that aortic wall degeneration in patients with abdominal aortic aneurysm was associated with mCRP immunopositivity and associated aberrant signaling pathway activation; the extent of which could be confirmed and marked by the hsCRP expression and thus utilized as a prognostic indicator.

In 2018, Zhang et al. (29) produced and validated an ‘experimental’ sandwich ELISA kit utilizing available CRP-binding commercial antibodies and which effectively discriminated and bound selectively mCRP with a sensitivity of around 1 ng/mL. They went on to compare nCRP and mCRP plasma levels in small cohorts of patients with skin autoimmune conditions eczema, psoriasis and urticaria, finding only mCRP levels were raised (most significantly in urticaria) compared with normal healthy controls. In a cohort of patients with acute myocardial infarction, showed increased plasma expression of mCRP-detected using characterized antibodies [with a diagnostic accuracy of 85% confidence] and expression was associated with risk of mortality (30).

So far, a commercially produced kit for accurate and sensitive measurement of plasma mCRP has eluded investigators with even the excellent mCRP-specific binding antibodies of Potempa lacking the absolute sensitivity, but certainly this could represent a novel and superior biomarker of inflammation if and when it becomes available. Jakuszko *et al.* (25) as mentioned earlier, already showed that auto-mCRP antibodies correlated with lupus nephritis disease activity and effective treatment reduced their serum expression and that of associated inflammatory cytokines.

Given the evidence described above, it is likely that pharmacological agents that could either 1) prevent the dissociation of nCRP to mCRP *in vivo*; 2) block the binding and activity of mCRP on the cell surface or 3) ‘clear’ mCRP tissue deposits by solubilization, opsonization or phagocytosis; might have major clinical significance. So far, breakthroughs have been limited, however, Fujita et al. recently showed that mCRP-specific antibody clone 3C suppressed leukocyte infiltration and symptoms of rheumatoid arthritis in a proof-of-concept murine collagen antibody induced model as well as lupus nephritis symptoms in an MRL/MP-lpr/lpr lupus prone model (31). Similarly, Garcia-Lara et al. (20), found that Potempa’s anti mCRP-8C10 antibody was able to completely block mCRP-induced chronic memory loss in a murine model of dementia where mCRP was stereotactically injected into the hippocampus resulting in symptoms of neurodegeneration. These preliminary data suggest that manipulation of mCRP by blocking its cellular signaling activation pathways could be an effective therapeutic approach.

An alternative approach is to identify or synthesize molecules with the ability to prevent nCRP dissociation mediated by phospholipase A2-dependent exposure to lysophosphatidylcholine at the surface of cell membranes. In this regard, Pepys et al. first demonstrated that 1, 6-bis(phosphocholine)-hexane was a small molecule capable of binding in a ration of 5:2 with individual sub-units of CRP; on binding, and applied to a rat model of myocardial infarction, both myocardial infarction volume and cardiac dysfunction were abrogated (32). Thiele et al. (5), *in vitro*, and *in vivo*, went on to demonstrate a significant reduction in inflammation and tissue deposition of mCRP in a rat model of myocardial infarction in the presence of this small molecule. An X-ray crystal structure of the pCRP-bis(PC)-H drug complex showed that five palindromic drug molecules were required to bind to phosphocholine sites and this created a linkage between two pentamers through crosslinking and occlusion of the ligand-binding B face, preventing dissociation.

This interaction abrogates binding of pCRP to known ligands such as LDL and blocks CRP-mediated complement C1q activation. Recently, Caprio et al. (33) and Slevin et al. (34), further discussed the probable structure of such small molecule inhibitors. Investigations have identified amino acid–ligand interactions and a crystal structure of pCRP bound to phosphocholine revealing key amino acids involved in ligand binding. Notably, the presence of a hydrophobic cavity adjacent to the binding region, is a prime target site for the design of inhibitors of pCRP–phosphocholine binding; with orphan drugs including acetylcholine providing *in vitro* evidence of prevention of mCRP-membrane binding with abrogation of inflammatory associated biology. Caution should be employed when considering therapeutic manipulation of a molecule such as CRP to ensure that the important normal physiological functions of the parent pentamer such as complement activation and opsonin protective characteristics remain. For this reason, the ideal therapeutic might be one that simply abrogates the dissociation thereby maintaining or in fact increasing the amount of circulating anti-inflammatory CRP whilst reducing the pro-inflammatory mCRP.

## Conclusion and Future Perspectives

CRP has now proven itself not only to be a marker of inflammation (in its native form) but more importantly, an important driver of the process and initiator of signal transduction cascades associated with pathologies linking to neurodegeneration, unstable atherosclerosis and autoimmune conditions amongst others. Modulation of mCRP might help protect from development of dementia, stroke and heart disease, mediated by chronic inflammatory-associated tissue damage. Over the coming years it would be anticipated that more pathologies harbor critical associations with mCRP deposition will be identified and characterized. Hence, strategies for therapeutic stabilization of native CRP thereby preventing its dissociation and/or CRP-binding proteins that block its cellular binding should be sought with urgency, whilst formulation of commercial technology enabling accurate measurement of this protein in the plasma should be prioritized.

## Author Contributions

All authors listed have made a substantial, direct, and intellectual contribution to the work and approved it for publication.

## Conflict of Interest

The authors declare that the research was conducted in the absence of any commercial or financial relationships that could be construed as a potential conflict of interest.

## Publisher’s Note

All claims expressed in this article are solely those of the authors and do not necessarily represent those of their affiliated organizations, or those of the publisher, the editors and the reviewers. Any product that may be evaluated in this article, or claim that may be made by its manufacturer, is not guaranteed or endorsed by the publisher.

## References

[1] Ngwa DN Agrawal A. Structure-Function Relationships of C-Reactive Protein in Bacterial Infection. Front Immunol (2019) 10:66. doi: 10.3389/fimmu.2019.00166 30863393 PMC6400226

[2] Sproston NR Ashworth JJ. Role of C-Reactive Protein at Sites of Inflammation and Infection. Front Immunol (2018) 9:754. doi: 10.3389/fimmu.2018.00754 29706967 PMC5908901

[3] Zeinolabediny Y Kumar S Slevin M. Monomeric-C-Reactive Protein – A Feature of Inflammatory Disease Associated With Cardiovascular Pathophysiological Complications? Vivo (2021) 34:693–7. doi: 10.21873/invivo.12309 PMC8045056 33622861

[4] Potempa LA Rajab M Olson ME Bordon J Fernandez-Botran R. C-Reactive Protein and Cancer: Interpreting the Differential Bioactivities of Its Pentameric and Monomeric, Modified Isoforms. Front Immunol (2021) 12:744129. doi: 10.3389/fimmu.2021.744129 34552600 PMC8450391

[5] Thiele JR Habersberger J Braig D Schmidt Y Goerendt K Maurer V. Dissociation of Pentameric to Monomeric C-Reactive Protein Localizes and Aggravates Inflammation. Circulation (2014) 130(1):35–50. doi: 10.1161/CIRCULATIONAHA.113.007124 24982116

[6] Cimmino G Loffredo FS Morello A D'Elia S De Palma R Cirillo P Golino P. Immune-Inflammatory Activation in Acute Coronary Syndromes: A Look Into the Heart of Unstable Coronary Plaques. Curr Cardiol Rev (2017) 13(2):110–7. doi: 10.2174/1573403X12666161014093812 PMC5452145 27758696

[7] Krupinski J Miguel-Turu M Martinez-Gonzalez J Carvajal A Juan-Babot JO Iborra E. Endogenous Expression of C-Reactive Protein Is Increased in Active (Ulcerated Noncomplicated) Human Carotid Artery Plaques. Stroke (2006) 37(5):1200–4. doi: 10.1161/01.STR.0000217386.37107.be 16601222

[8] Badimon L Pena E Arderiu G Padró T Slevin M Vilahur G. C-Reactive Protein in Atherothrombosis and Angiogenesis. Front Immunol (2018) 9:430. doi: 10.3389/fimmu.2018.00430 29552019 PMC5840191

[9] Molins B Pena E Vilahur G Mendieta C Slevin M Badimon L. C-Reactive Protein Isoforms Differ in Their Effects on Thrombus Growth. Arterioscler Thromb Vasc Biol (2008) 28(12):2239–46. doi: 10.1161/ATVBAHA.108.174359 18787187

[10] Sun H Koike T Ichikawa T Hatakeyama K Shiomi M Zhang B. C-Reactive Protein in Atherosclerotic Lesions. Am J Pathol (2005) 167(4):1139–48. doi: 10.1016/S0002-9440(10)61202-3 PMC1603667 16192648

[11] Siennicka A. Association Between Microvesicles Bearing Monomeric-C-Reactive Protein and Platelet Reactivity. Relationship With Low Response to Antiplatelet Drugs? J Physiol Pharmacol (2021) 72(1):3–12. doi: 10.26402/jpp.2021.1.01 34099580

[12] Habersberger J Strang F Scheichl A Htun N Bassler N Merivirta RM. Circulating Microparticles Generate and Transport Monomeric C-Reactive Protein in Patients With Myocardial Infarction. Cardiovasc Res (2012) 96(1):64–72. doi: 10.1093/cvr/cvs237 22798388

[13] Melnikov I Kozlov S Saburova O Zubkova E Guseva O Domogatsky S. CRP Is Transported by Monocytes and Monocyte-Derived Exosomes in the Blood of Patients With Coronary Artery Disease. Biomedicines (2020) 8(10):435. doi: 10.3390/biomedicines8100435 PMC7589628 33086769

[14] Zha Z Cheng Y Cao L Qian Y Liu X Guo Y. Monomeric CRP Aggravates Myocardial Injury After Myocardial Infarction by Polarizing the Macrophage to Pro-Inflammatory Phenotype Through JNK Signaling Pathway. J Inflamm Res (2021) 14:7053–64. doi: 10.2147/JIR.S316816 PMC8703048 34984018

[15] Yu Q Yang P Wang Y Wang Y Zhao S Yang P. C-Reactive Protein Is Associated With the Progression of Acute Embolic Stroke in Rabbit Model. J Thromb Thrombolysis (2012) 33(4):301–7. doi: 10.1007/s11239-011-0627-0 21874402

[16] Slevin M Matou-Nasri S Turu M Luque A Rovira N Badimon L. Modified C-Reactive Protein Is Expressed by Stroke Neovessels and is a Potent Activator of Angiogenesis In Vitro. Brain Pathol (2010) 20(1):151–65. doi: 10.1111/j.1750-3639.2008.00256.x PMC8094831 19170684

[17] Slevin M Garcia-Lara E Capitanescu B Sanfeliu C Zeinolabediny Y AlBaradie R. Monomeric-C-Reactive Protein Aggravates Secondary Degeneration After Intracerebral Haemorrhagic Stroke and may Function as a Sensor for Systemic Inflammation. J Clin Med (2020) 22(9):3053. doi: 10.3390/jcm9093053 PMC7563733 32971821

[18] Strang F Scheichl A Chen YC Wang X Hyun NM Bassler N. Amyloid Plaques Dissociate Pentameric to Monomeric-C-Reactive Protein: A Novel Pathomechanism Driving Cortical Inflammation in Alzheimer’s Disease? Brain Pathol (2012) 22(3):337–46. doi: 10.1111/j.1750-3639.2011.00539.x PMC8092962 21951392

[19] Slevin M Matou S Zeinolabediny Y Corpas R Weston R Liu D. Monomeric C-Reactive Protein-a Key Molecule Driving Development of Alzheimer’s Disease Associated With Brain Ischaemia? Sci Rep (2015) 5:13281. doi: 10.1038/srep13281 26335098 PMC4558604

[20] Garcia-Lara E Aguirre S Clotet N Sawkulycz X Bartra C Almenara-Fuentes L. Antibody Protection Against Long-Term Memory Loss Induced by Monomeric C-Reactive Protein in a Kouse Model of Dementia. Biomedicines (2021) 9(7):828. doi: 10.3390/biomedicines9070828 34356892 PMC8301488

[21] Al-Baradie R Pu S Liu D Zeinolabediny Y Ferris G Sanfeliu C. Monomeric C-Reactive Protein Localised in the Cerebral Tissue of Damaged Brain Regions Is Associated With Neuro-Inflammation and Neurodegeneration-An Immunohistochemical Study. Front Immunol (2021) 12:644213. doi: 10.3389/fimmu.2021.644213 33796111 PMC8007856

[22] Zhang Z Na H Gan H Tao Q Alekseyev Y Hu J. Monomeric C-Reactive Protein *via* Endothelial CD31 for Neurovascular Inflammation in an ApoE Genotype-Dependent Pattern: A Risk Factor for Alzheimer’s Disease? Aging Cell (2021) 20(11):e13501. doi: 10.1111/acel.13501 34687487 PMC8590103

[23] Romero-Vazquez Adan A Figueras-Roca M Llorenç V Slevin M Vilahur G. Activation of C-Reactive Protein Proinflammatory Phenotype in the Blood Retinal Barrier *In Vitro*: Implications for Age-Related Macular Degeneration. Aging (2020) 12(14):13905–23. doi: 10.18632/aging.103655 PMC7425453 32673285

[24] Fendl B Weiss R Eichhorn T Linsberger I Afonyushkin T Puhm F. Extracellular Vesicles are Associated With C-Reactive Protein in Sepsis. Sci Rep (2021) 11(1):m6996. doi: 10.1038/s41598-021-86489-4 PMC7997920 33772103

[25] Jakuszko K Krajewska M Koscielska-Kasprzak K Myszka M Sebastian A Gniewek K. Antibodies Against Monomeric C-Reactive Protein-a Promising Biomarker of Lupus Nephritis? Clin Biochem (2017) 50(13):756–62. doi: 10.1016/j.clinbiochem.2017.03.010 28300543

[26] Ruiz-Fernandez C Gonzalez-Rodriguez M Francisco V Rajab IM Gómez R Conde J. Monomeric C-Reactive Protein (mCRP) Regulates Inflammatory Responses in Human and Mouse Chondrocytes. Lab Invest (2021) 101(12):1550–60. doi: 10.1038/s41374-021-00584-8 33767361

[27] Zeller J Bogner B Kiefer J Braig D Winninger O Fricke M. CRP Enhances the Innate Killing Mechanisms Phagocytosis and ROS Formation in a Conformation and Complement-Dependent Manner. Front Immunol (2021) 12:721887. doi: 10.3389/fimmu.2021.721887 34447388 PMC8383111

[28] Kim EN Yu J Lim JS Jeong H Kim CJ Choi JS. CRP Immune-Deposition and Proteomic Analysis in Abdominal Aortic Aneurysm. PloS One (2021) 16(8):e0245361. doi: 10.1371/journal.pone.0245361 34428207 PMC8384196

[29] Zhang L Li HY Shen ZY Shen ZY Wang YD Ji SR. An ELISA Assay for Quantifying Monomeric C-Reactive Protein in Plasma. Front Immunol (2018) 9:511. doi: 10.3389/fimmu.2018.00511 29593741 PMC5857914

[30] Wang J Tang B Liu X Wang J Tang B Liu X. Increased Monomeric CRP Levels in Acute Myocardial Infarction: A Possible New and Specific Biomarker for Diagnosis and Severity Assessment of Disease. Atherosclerosis (2015) 239(2):343–9. doi: 10.1016/j.atherosclerosis.2015.01.024 25682033

[31] Fujita C Sakurai Y Yasuda Y Takada Y Huang CL Fujita M. Anti-Monomeric C-Reactive Protein Antibody Ameliorates Arthritis and Nephritis in Mice. J Immunol (2021) 207(7):1755–62. doi: 10.4049/jimmunol.2100349 34470853

[32] Pepys MB Hirshfield GM Tennent GA Gallimore JR Kahan MC Bellotti V. Targeting C-Reactive Protein for the Treatment of Cardiovascular Disease. Nature (2006) 440(7088):1217–21. doi: 10.1038/nature04672 16642000

[33] Caprio V Badimon L Di Napoli M Fang WH Ferris GR Guo B. pCRP-mCRP Dissociation Mechanism as Potential Targets for the Development of Small-Molecule Anti-Inflammatory Chemotherapeutics. Front Immunol (2018) 9:1089. doi: 10.3389/fimmu.2018.01089 29892284 PMC5985513

[34] Slevin M Iemma RS Zeinolabediny Y Liu D Ferris GR Caprio V. Acetylcholine Inhibits Monomeric C-Reactive Protein Induced Inflammation, Endothelial Cell Adhesion and Platelet Aggregation; a Potential Therapeutic? Front Immunol (2018) 9:2124. doi: 10.3389/fimmu.2018.02124 30319609 PMC6168760

